# Supplementary material for: Comparing enzyme activity modifier equations through the development of global data fitting templates in Excel
Source: PeerJ. 2018 Dec 14;6:e6082. doi: 10.7717/peerj.6082 (PMC6296338; doi:10.7717/peerj.6082)
Supplement: Supplemental Information 1 [file peerj-06-6082-s001.docx]

***Derivation of the substrate affinity modification equation***

The method of using reaction schemes to generate the traditional inhibition equations can also be used to derive the components of the modifier equation. The modifier term affecting the V_max_ is a rearrangement of the Non-competitive equation so directly comes from reaction schemes (Walsh 2012). The modifier term affecting the K_M_ is likewise definable from an appropriate reaction scheme from which the rate and conservation of mass equations are used to derive the expression. For this derivation, it must be assumed that binding of inhibitor or modifier only affects the affinity of the substrate for the enzyme and does not prevent substrate hydrolysis. For simplicity, the following equations will use “I” to represent the inhibitor concentration although they could just as easily use “X” for modifier concentration. This results in a rate equation that considers total substrate hydrolysis by the enzyme population, both bound by inhibitor and unbound.

$$v=k_{cat}\left[ ES \right]+k_{cat}\left[ EIS \right]$$

For additional simplicity, the conservation of mass equation will group the portion of the enzyme population not involved in substrate hydrolysis, the enzyme alone ([E]) and the enzyme-inhibitor complex ([EI]) as ([E’]).

$$E_{tot}=\left[ E \right]+\left[ EI \right]+\left[ ES \right]+\left[ EIS \right]$$

$$E_{tot}=\left[ E' \right]+\left[ ES \right]+\left[ EIS \right]$$

Additionally, equilibrium constants for the substrate and inhibitor are defined.

$$\left[ ES \right]=\frac{\left[ E' \right]\left[ S \right]}{K_{M}}$$

$$\left[ EIS \right]=\frac{\left[ ES \right]\left[ I \right]}{K_{i}}$$

Where the substrate affinity (K_M_) is assumed to be an equilibrium constant for the substrate disassociation from the substrate-enzyme complex and the enzyme substrate modifier complex as proposed by Fontes et al., 2000.

The derivation can then be initiated by multiplying the rate equation by 1, which in this case takes the form of E_tot_ divided by its parts.

$$v=k_{cat}\left[ ES \right]+k_{cat}\left[ EIS \right]\times\frac{E_{tot}}{[E']+[ES]+[EIS]}$$

$$\frac{v}{E_{tot}}=\frac{k_{cat}\left[ ES \right]+k_{cat}\left[ EIS \right]}{[E']+[ES]+[EIS]}$$

This equation can then be rearranged by substituting for the enzyme-substrate complex (ES) and enzyme-inhibitor-substrate complex (EIS).

$$\frac{v}{E_{tot}}=\frac{k_{cat}\left( \frac{\left[ E' \right]\left[ S \right]}{K_{M}} \right)+k_{cat}\left( \frac{\left[ ES \right]\left[ I \right]}{K_{i}} \right)}{[E']+\left( \frac{\left[ E' \right]\left[ S \right]}{K_{M}} \right)+\left( \frac{\left[ ES \right]\left[ I \right]}{K_{i}} \right)}$$

This is repeated by resubstituting for the enzyme-substrate complex (ES).

$$\frac{v}{E_{tot}}=\frac{k_{cat}\left( \frac{\left[ E' \right]\left[ S \right]}{K_{M}} \right)+k_{cat}\left( \frac{\left[ E' \right]\left[ S \right]\left[ I \right]}{K_{M}K_{i}} \right)}{[E']+\left( \frac{\left[ E' \right]\left[ S \right]}{K_{M}} \right)+\left( \frac{\left[ E' \right]\left[ S \right]\left[ I \right]}{K_{M}K_{i}} \right)}$$

Factoring out free enzyme (E’).

$$\frac{v}{E_{tot}}=\frac{k_{cat}\left( \frac{\left[ S \right]}{K_{M}} \right)+k_{cat}\left( \frac{\left[ S \right]\left[ I \right]}{K_{M}K_{i}} \right)}{1+\left( \frac{\left[ S \right]}{K_{M}} \right)+\left( \frac{\left[ S \right]\left[ I \right]}{K_{M}K_{i}} \right)}$$

Multiplying by K_M_

$$\frac{v}{E_{tot}}=\frac{k_{cat}\left[ S \right]+k_{cat}\left[ S \right]\left( \frac{\left[ I \right]}{K_{i}} \right)}{K_{M}+\left[ S \right]+\left[ S \right]\left( \frac{\left[ I \right]}{K_{i}} \right)}$$

Factoring out the inhibitory term (1+[I]/Ki).

$$\frac{v}{E_{tot}}=\frac{k_{cat}\left[ S \right]\left( 1+\frac{\left[ I \right]}{K_{i}} \right)}{K_{M}+\left[ S \right]\left( 1+\frac{\left[ I \right]}{K_{i}} \right)}$$

Dividing by the inhibitory term

$$\frac{v}{E_{tot}}=\frac{k_{cat}\left[ S \right]}{\frac{K_{M}}{\left( 1+\frac{\left[ I \right]}{K_{i}} \right)}+\left[ S \right]}$$

Followed by rearranging into the typical MM form.

$$v=\frac{E_{tot}k_{cat}\left[ S \right]}{\frac{K_{M}}{\left( 1+\frac{\left[ I \right]}{K_{i}} \right)}+\left[ S \right]}$$

$$v=\frac{V_{max}\left[ S \right]}{\frac{K_{M}}{\left( 1+\frac{\left[ I \right]}{K_{i}} \right)}+\left[ S \right]}$$

Rearranging the inhibitory term leads to the generalized hyperbolic term for modifying substrate affinity.

$$v=\frac{V_{max}\left[ S \right]}{K_{M}\left( 1-\frac{\left[ I \right]}{{\left[ I \right]+K}_{i}} \right)+\left[ S \right]}$$

$$v= \frac{V_{max}\left[ S \right]}{K_{M}-K_{M}\frac{\left[ I \right]}{{\left[ I \right]+K}_{i}}+\left[ S \right]}$$

$$v= \frac{V_{max}\left[ S \right]}{K_{M}-{\Delta K}_{M}\frac{\left[ I \right]}{{\left[ I \right]+K}_{i}}+\left[ S \right]}$$

Which can be used to define modifiers that activate as well.

$$v= \frac{V_{max}\left[ S \right]}{K_{M}-{\Delta K}_{M}\frac{\left[ X \right]}{{\left[ X \right]+K}_{x}}+\left[ S \right]}$$
