# Supplementary material for: Comparing enzyme activity modifier equations through the development of global data fitting templates in Excel
Source: PeerJ. 2018 Dec 14;6:e6082. doi: 10.7717/peerj.6082 (PMC6296338; doi:10.7717/peerj.6082)

**Installing Solver in Excel.**

To fit data in the template, the Solver Add-in of Excel should be installed. In Excel 2016 the Add-ins can be found under the **File** tab of the Excel ribbon.


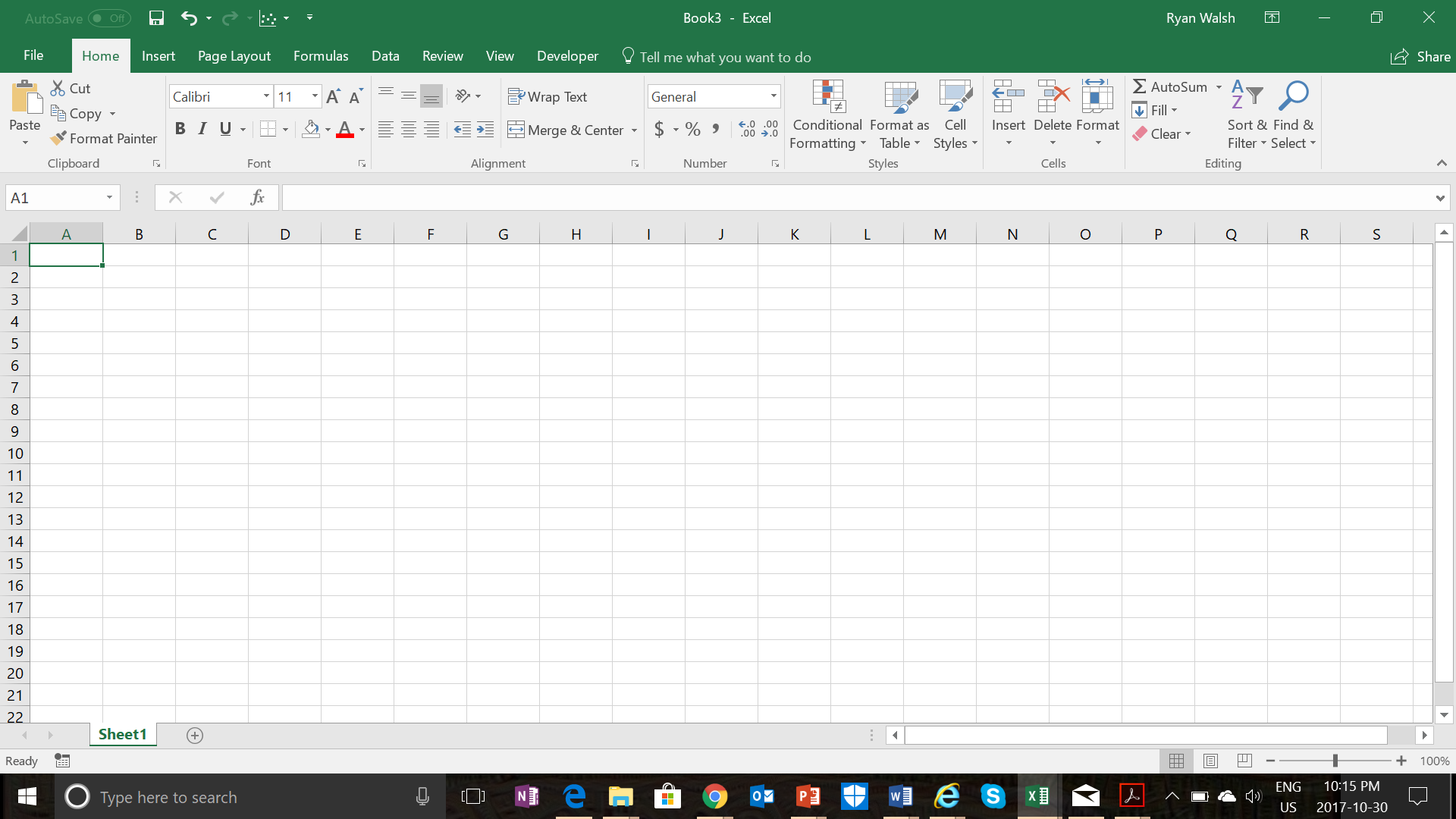


Under the File tab select **Options**.


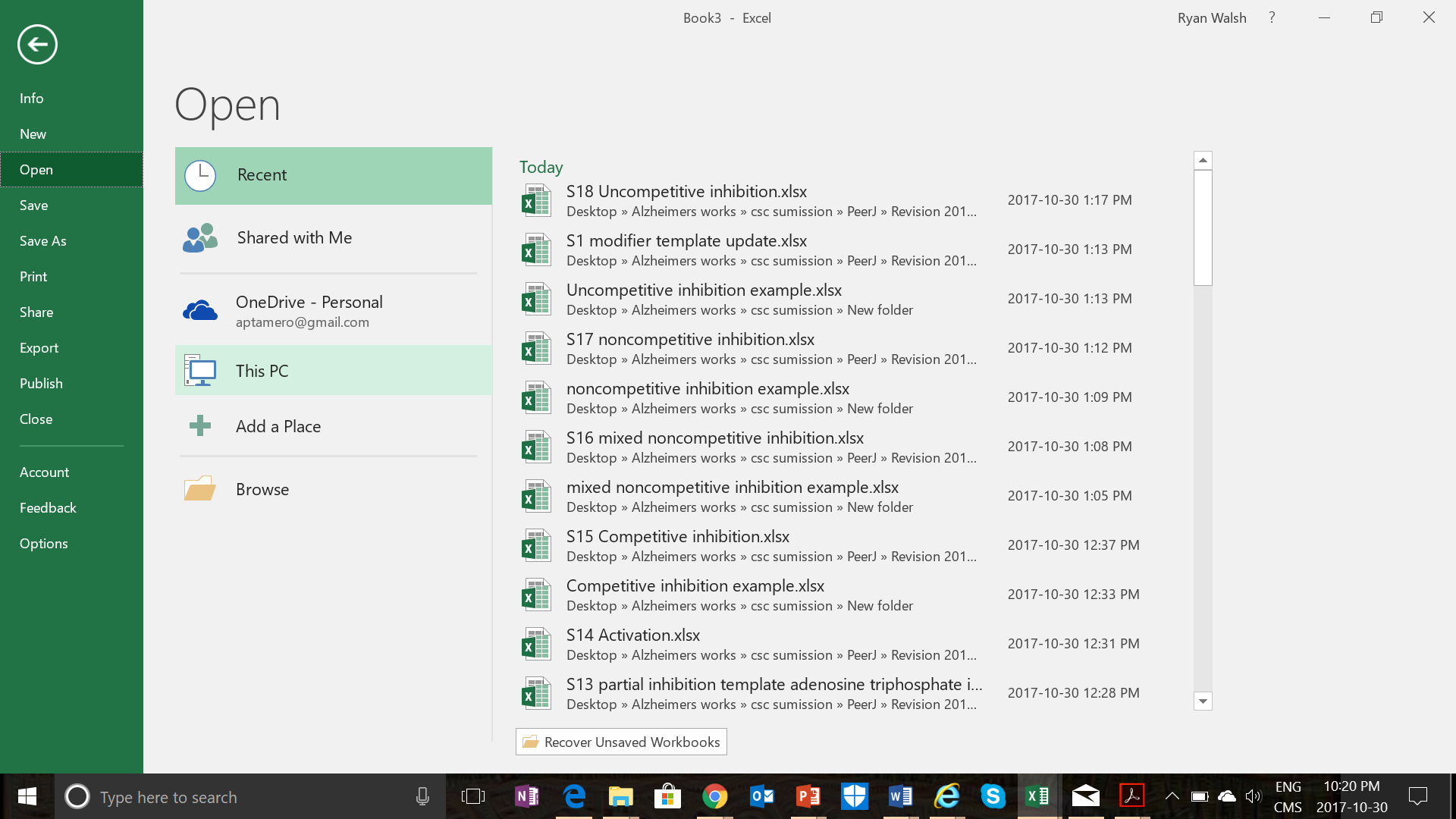


In the options select **Add-ins.**


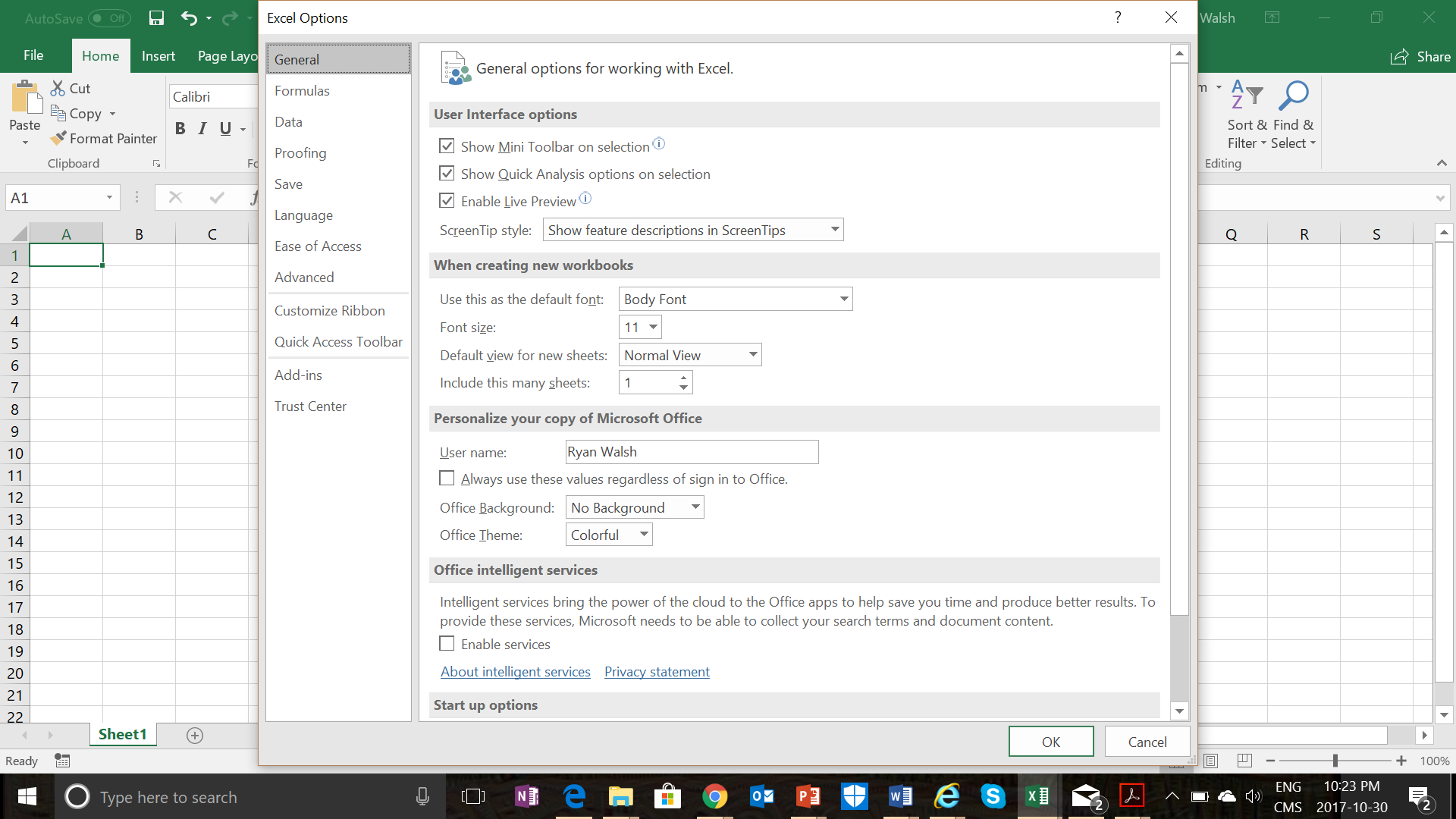


Next select **Go** next to manage Excel Add-ins.


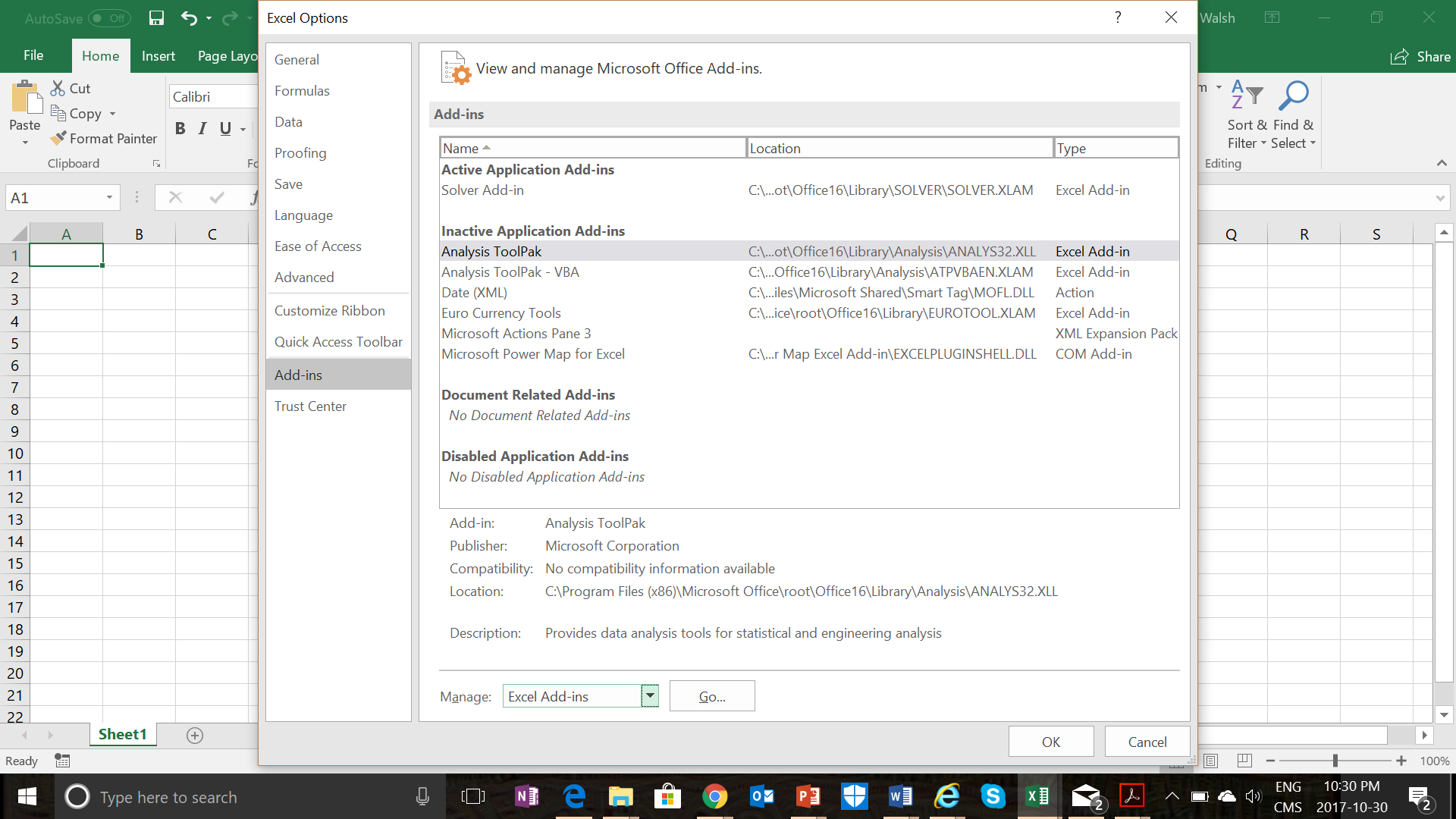


Check the Solver Add-in and click **OK.**


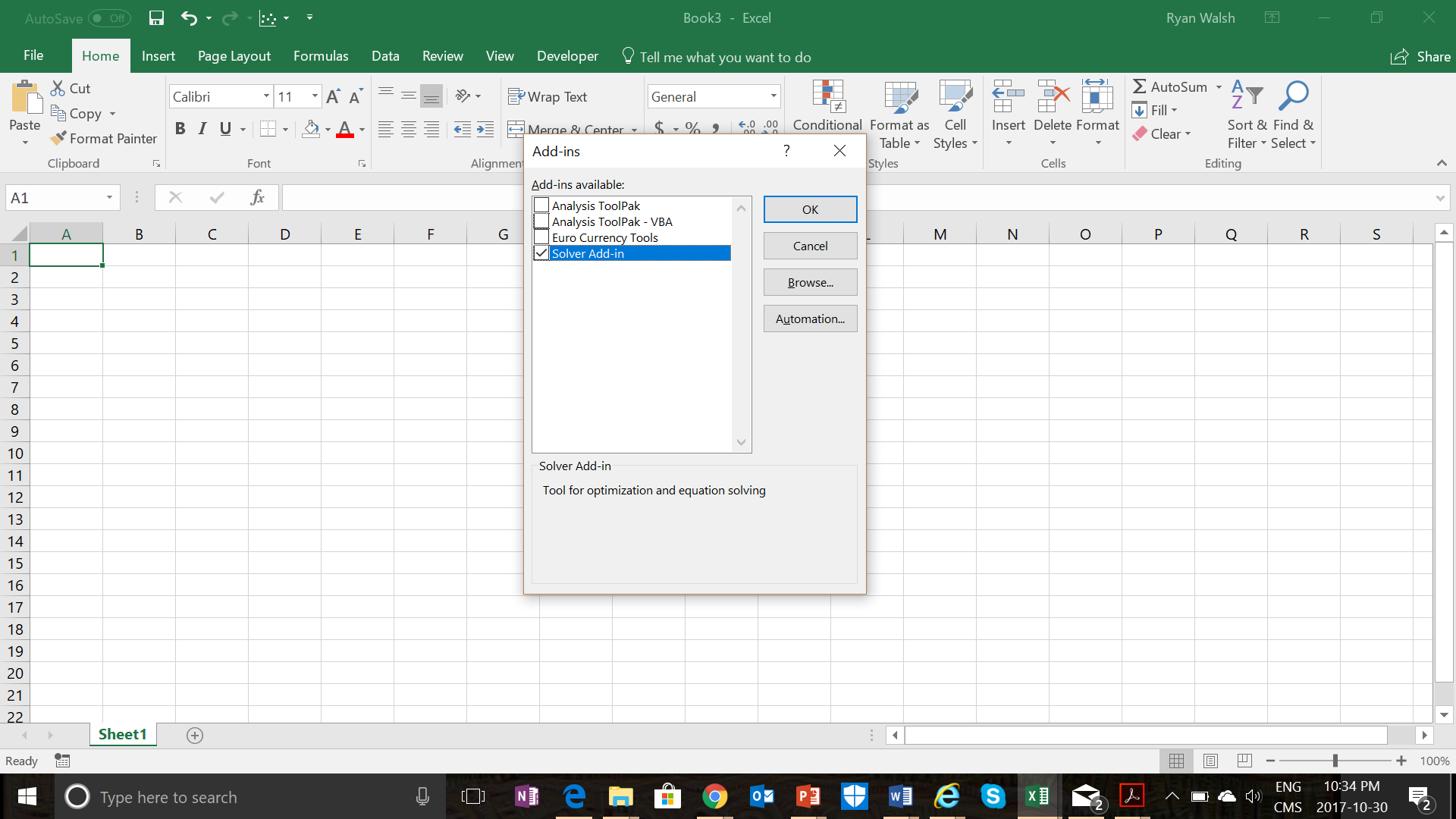


The solver Add-in should now be available in the data tab of the Excel ribbon.


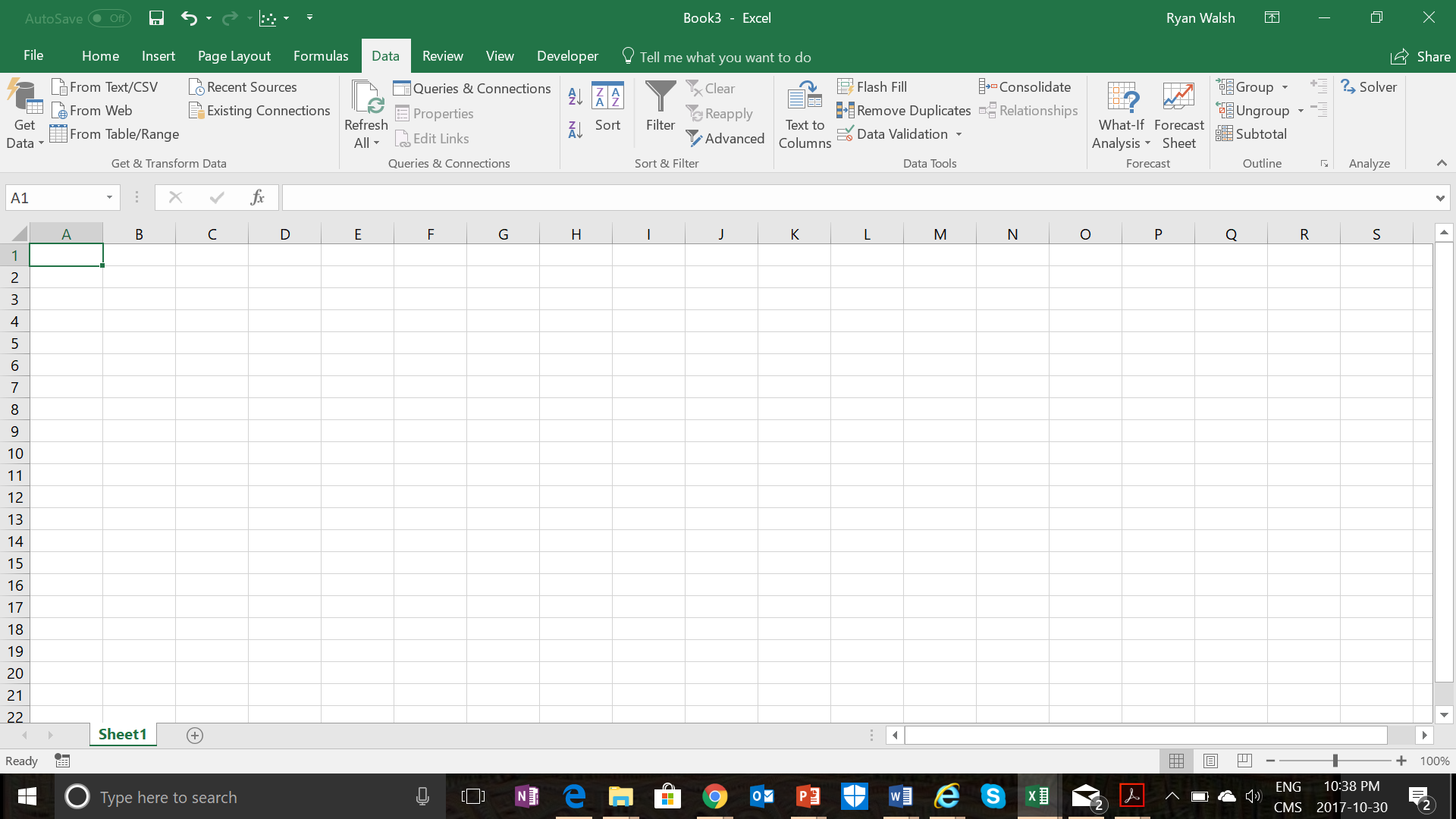


**Fitting Data in the template**

Insert the data to be fit into the first sheet of the spreadsheet, in the example below the simulated data from the uncompetitive inhibition supplementary data is displayed.


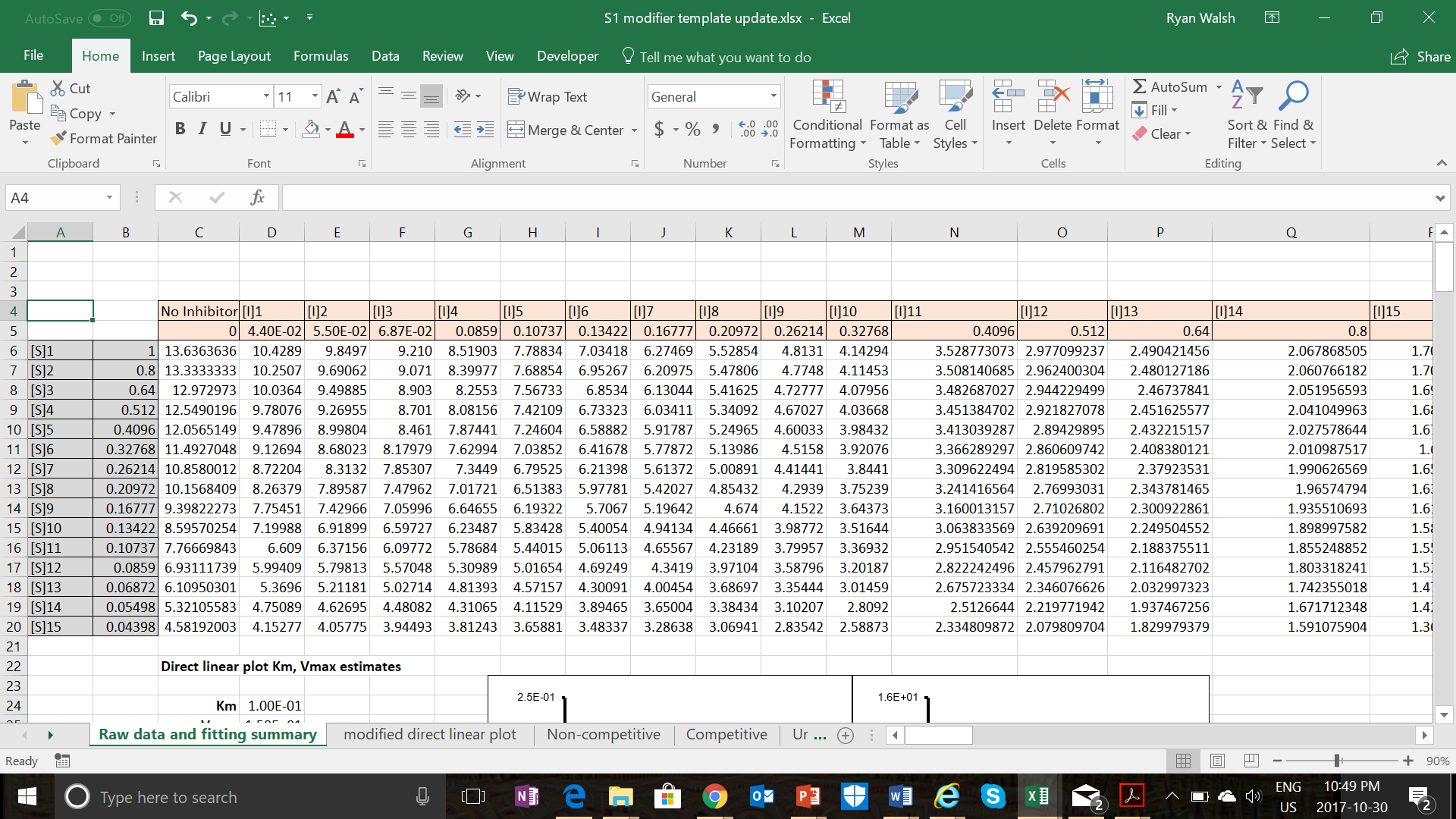


From the raw data, the template estimates the initial kinetic parameters for the global fitting using a modified direct linear plot for the K_M_ and V_max_ along with a K_i_ estimate based on a linear decrease in activity with increasing inhibitor concentrations.


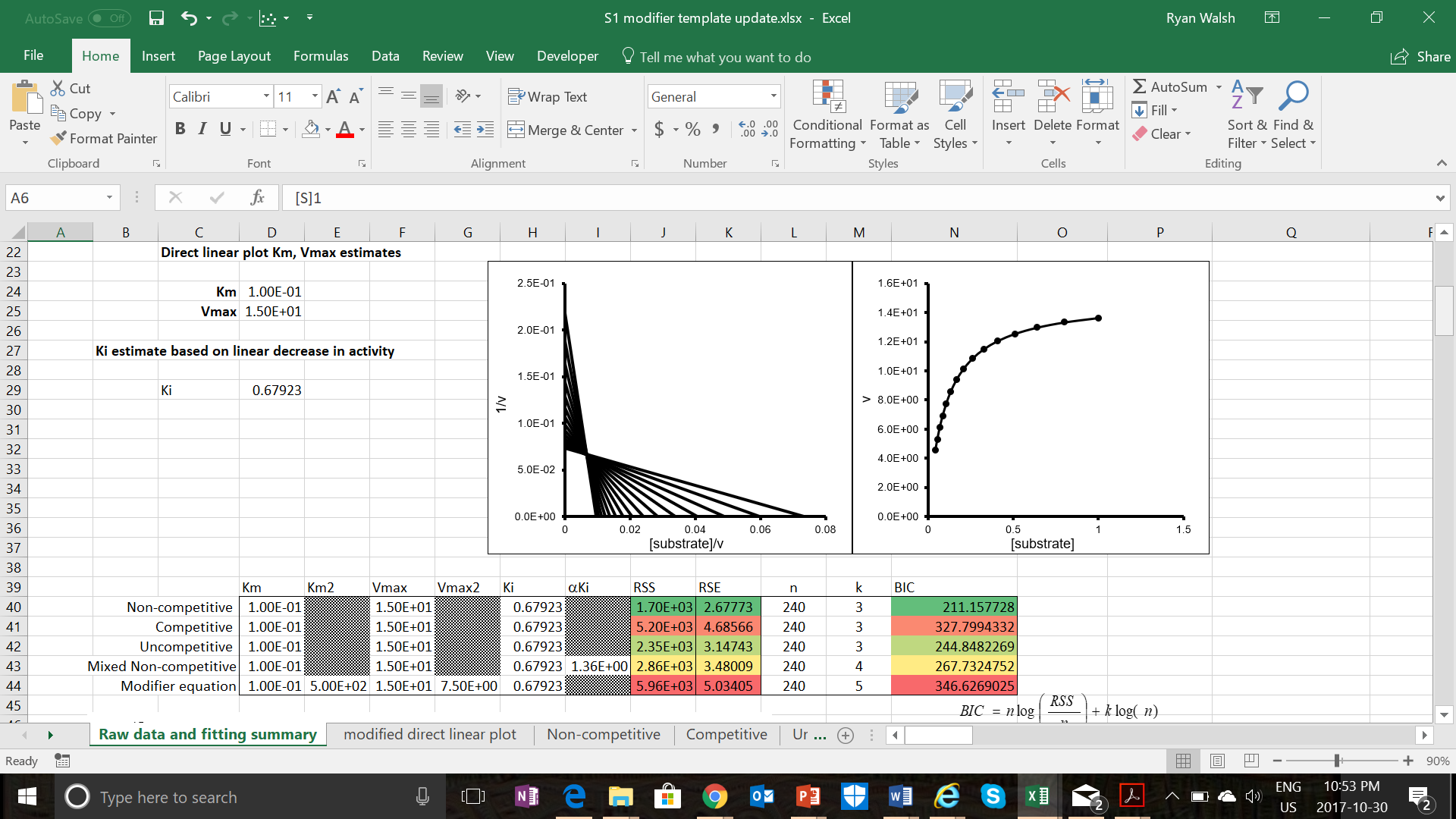


The additional values of K_m2_ and αK_i_ are just multiples of K_m_ and K_i_ respectively. If desired the initial values may be changed manually. These initial values provide a rough fitting of the data which can now be optimized using the Solver Add-in.


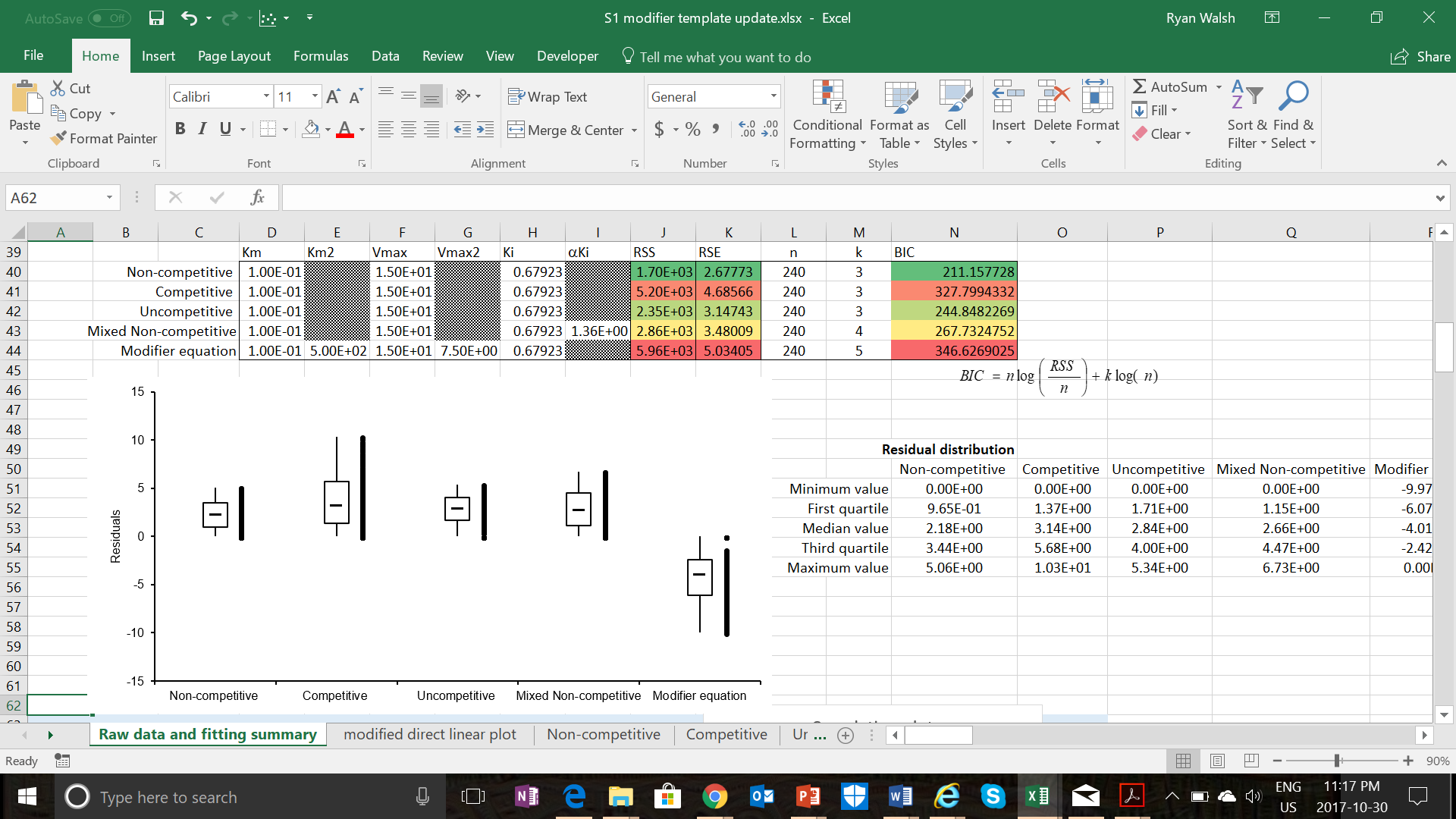


Open the Solver Add-in the Data tab and select the **objective,** which depending on the model to be fit would be the RSS value of that model (i.e., for the Non-competitive model select the RSS in J40).


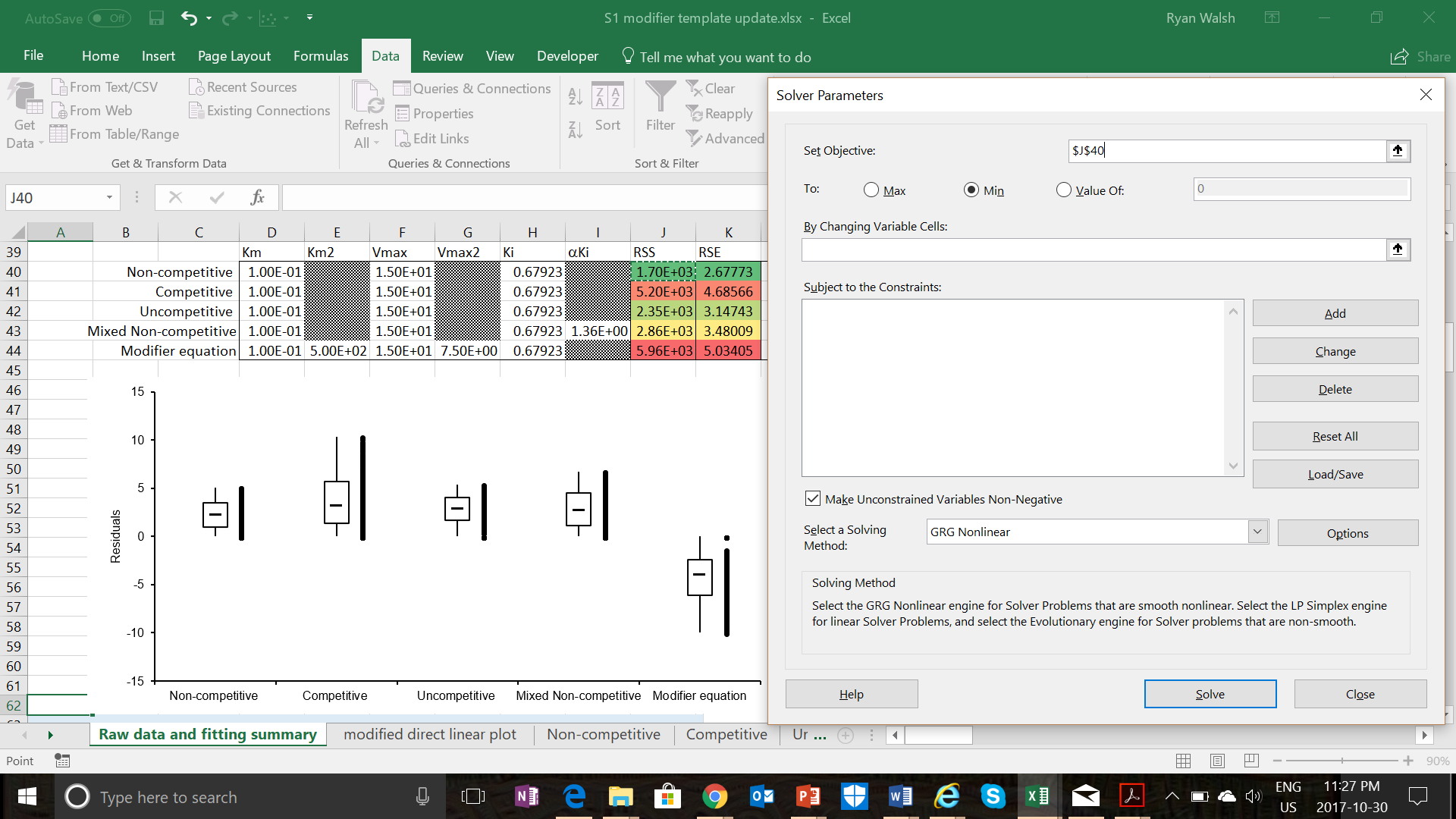


Make sure that the objective RSS value is going to be minimized.


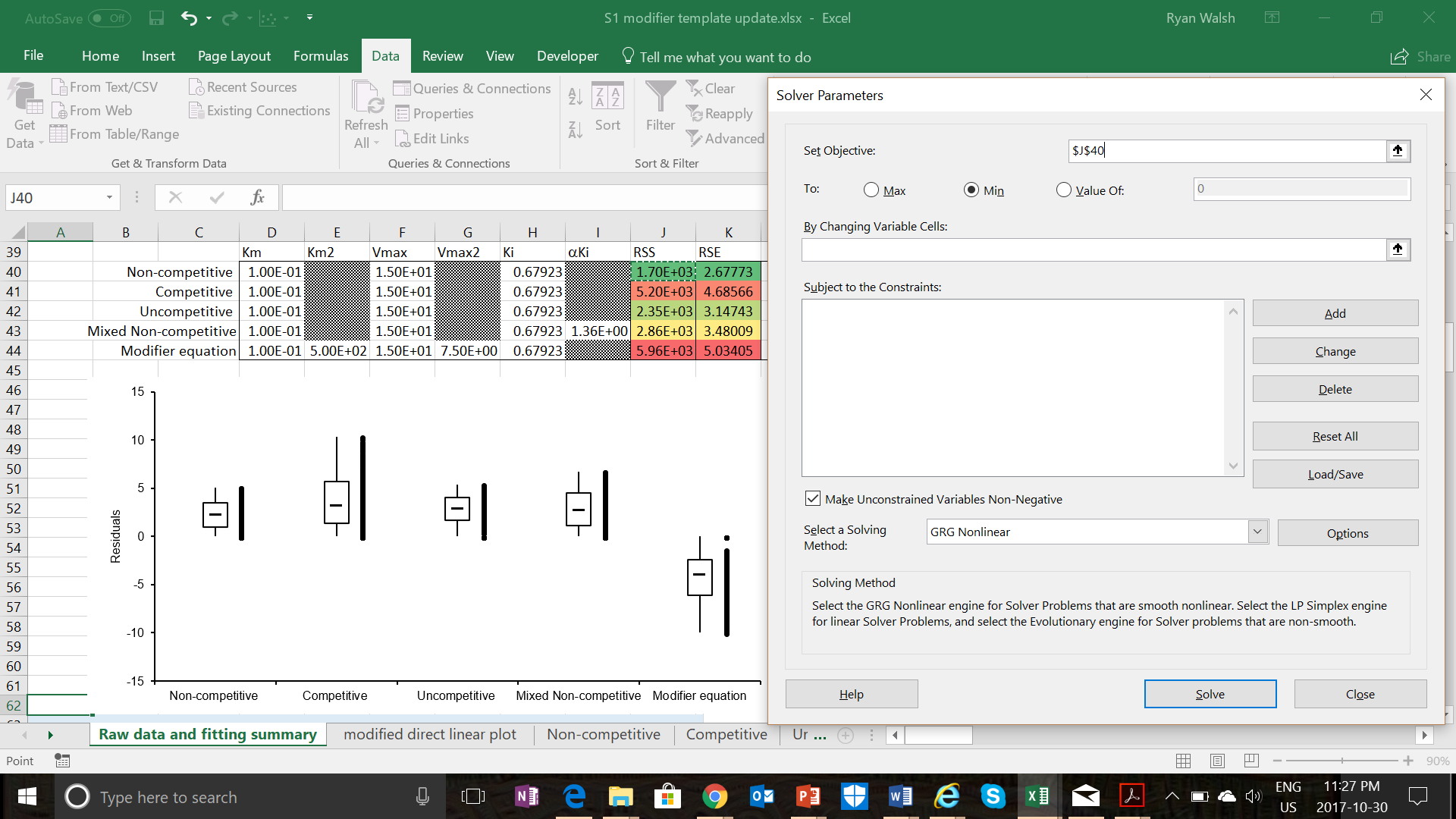


Then select the **variables to be changed**, in this case, the K_m_ V_max_ and K_i_ of the non-competitive model located in the cells D40, F40 and H40, then hit Solve.


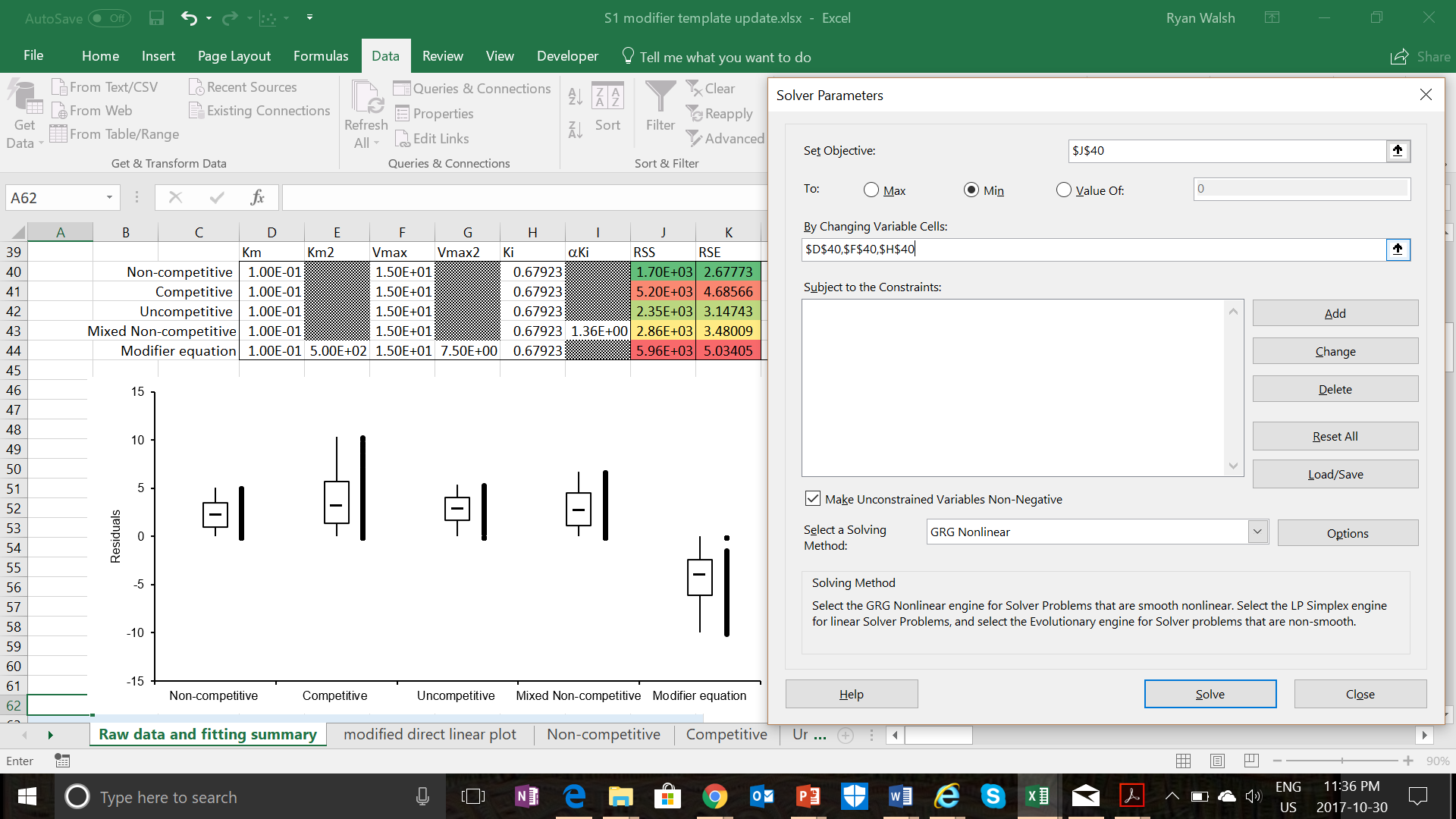


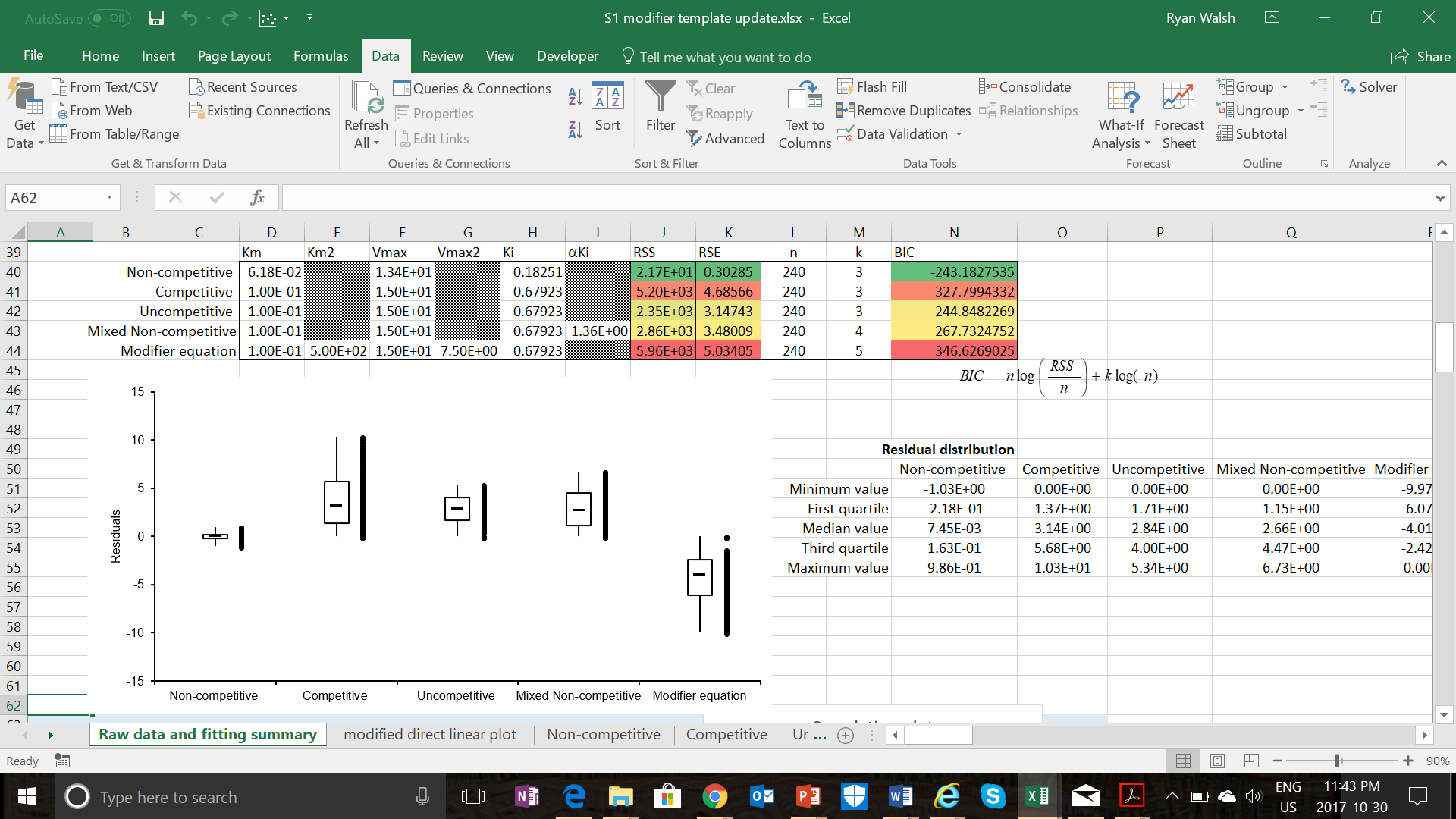


Repeat the fitting process with each model minimizing the RSS values by varying their respective kinetic parameters.


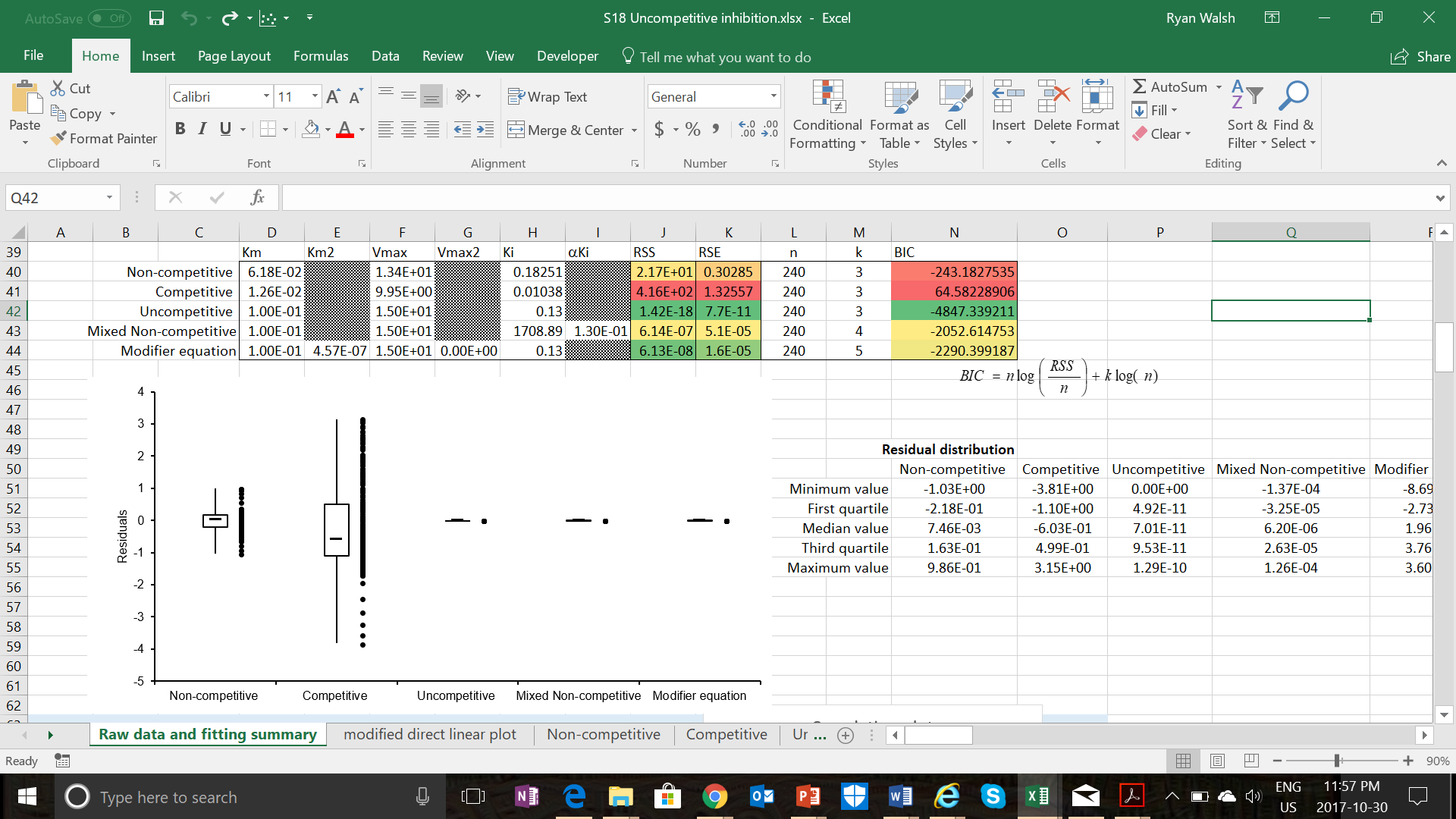

Supplement: Supplemental Information 3 [file peerj-06-6082-s003.docx]
